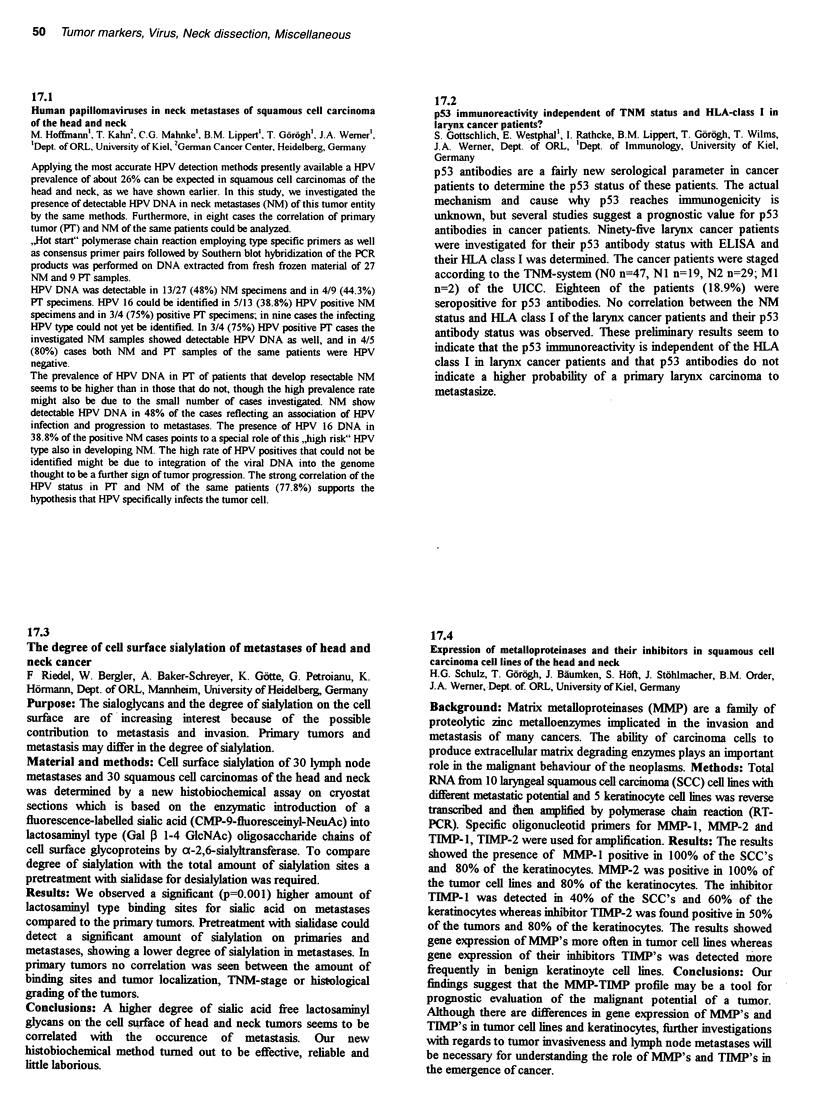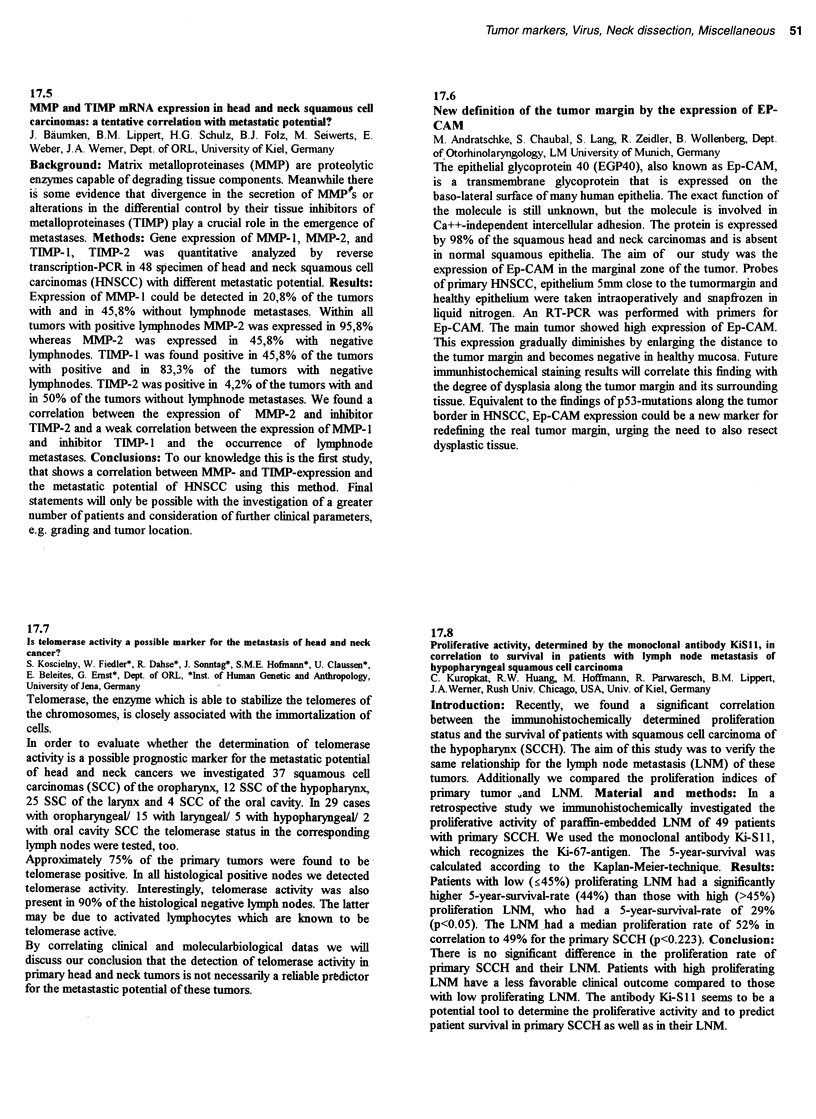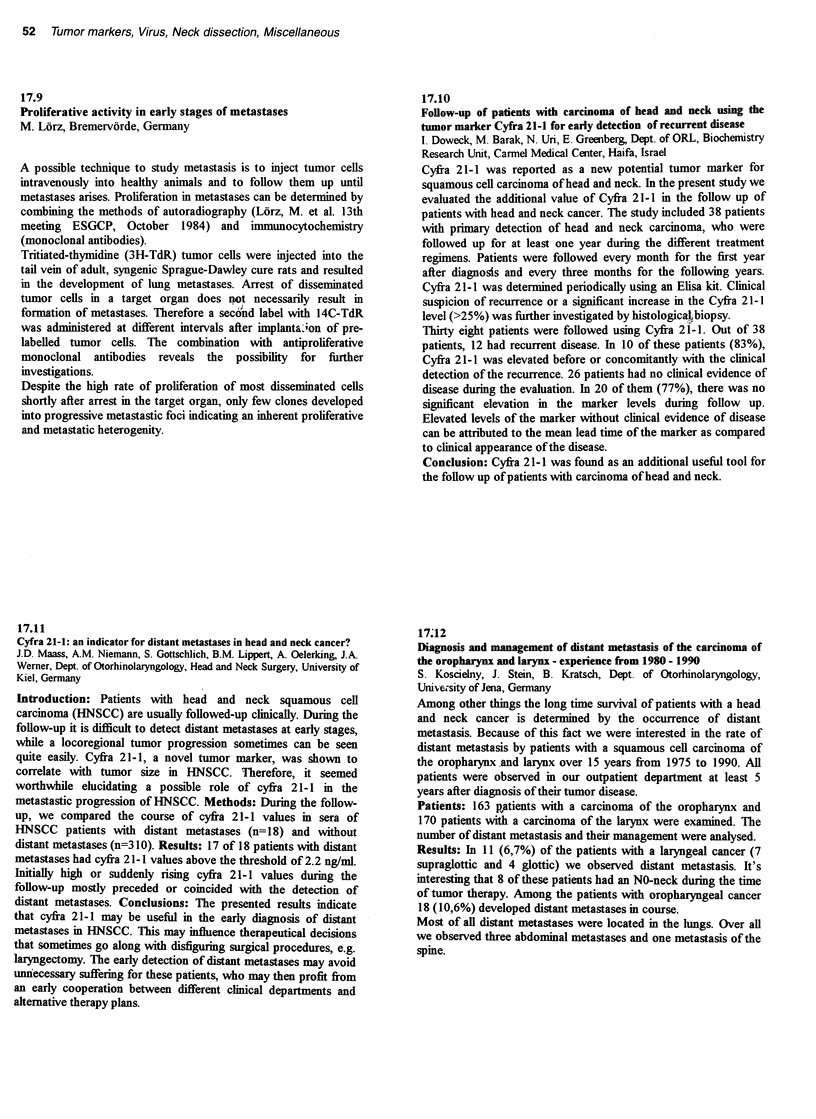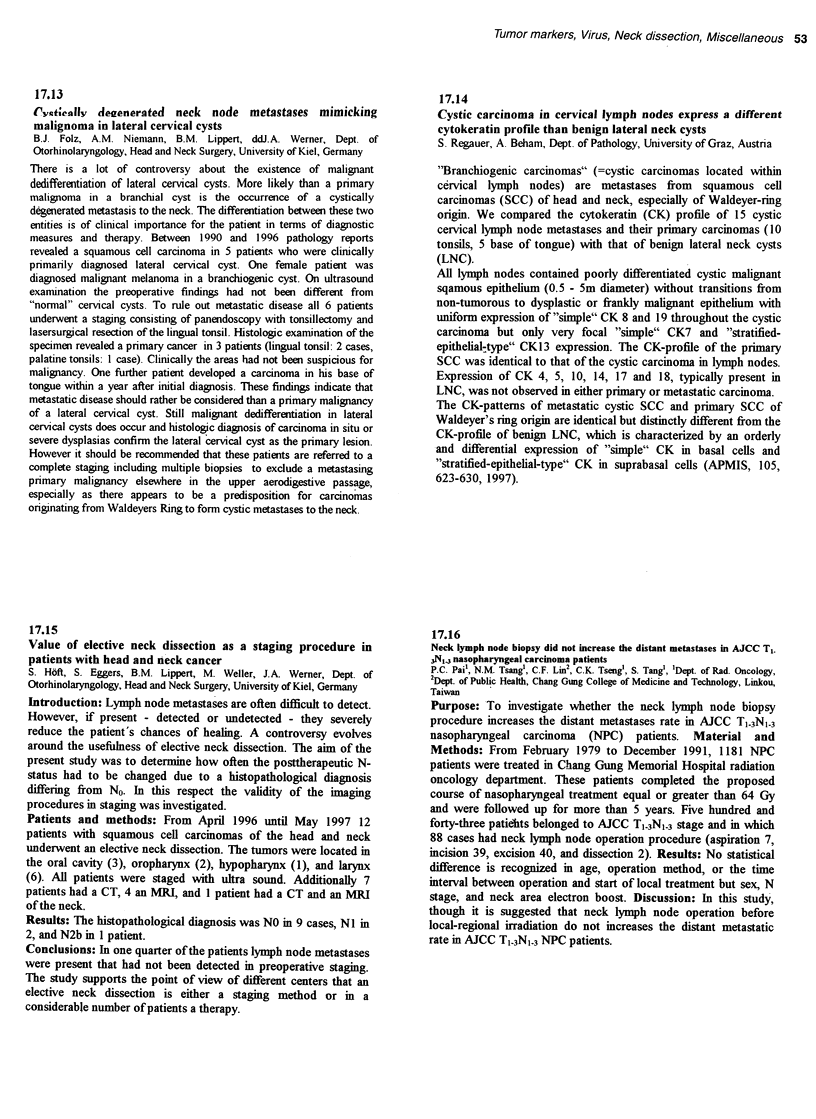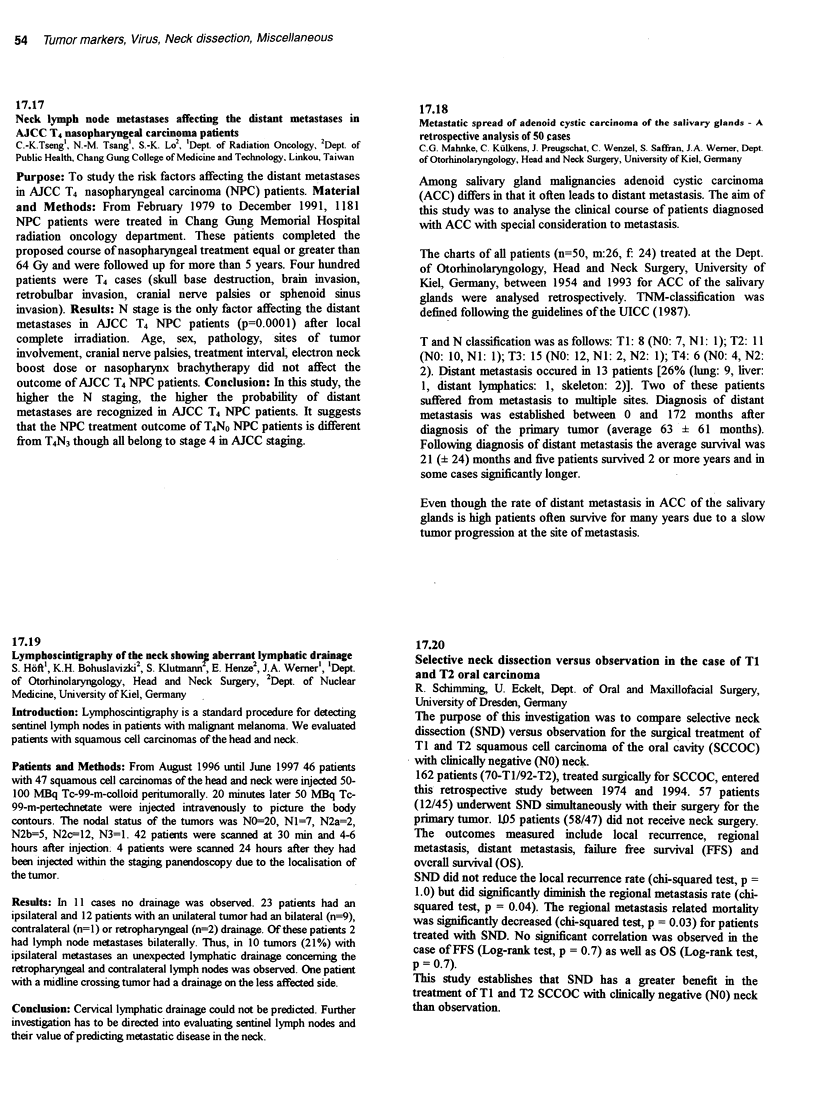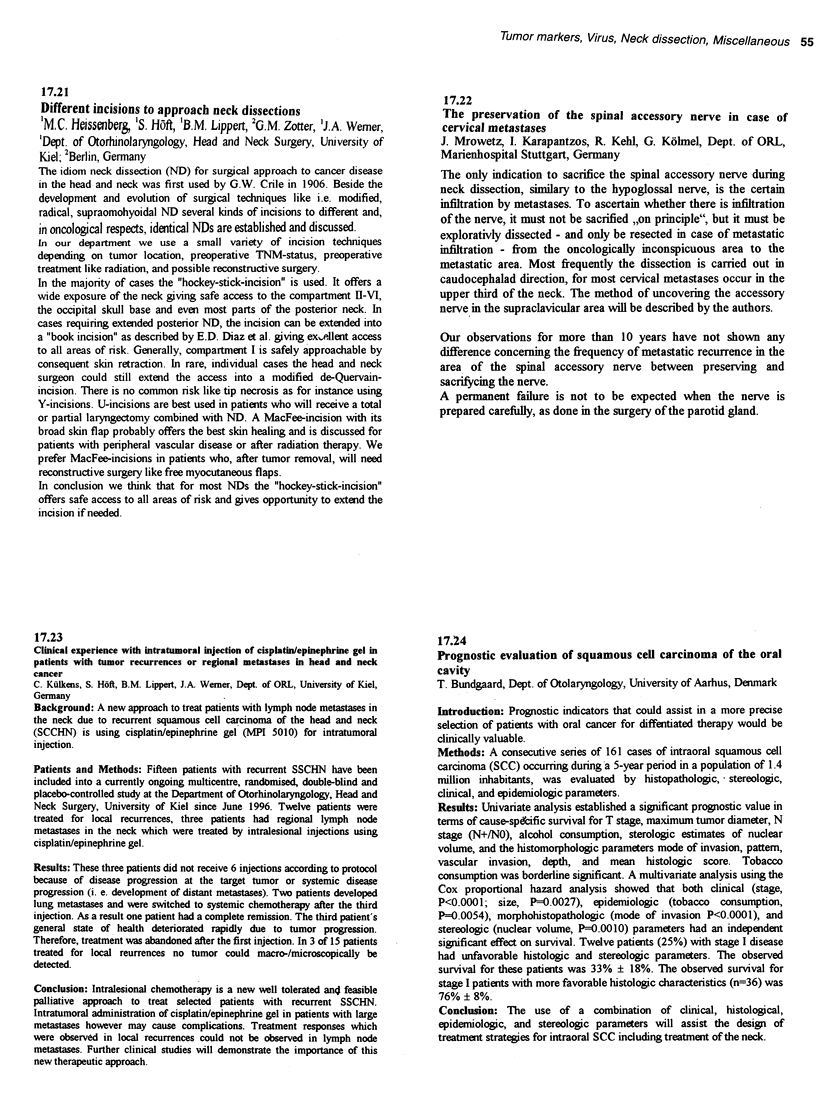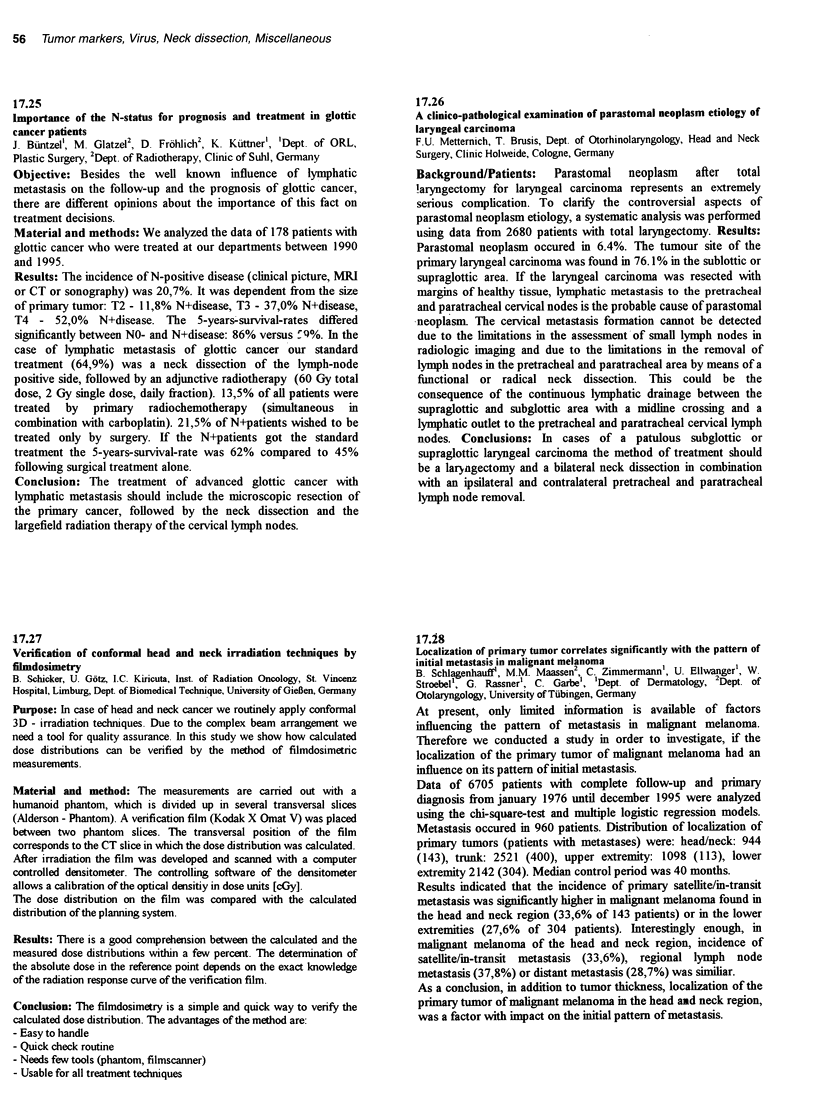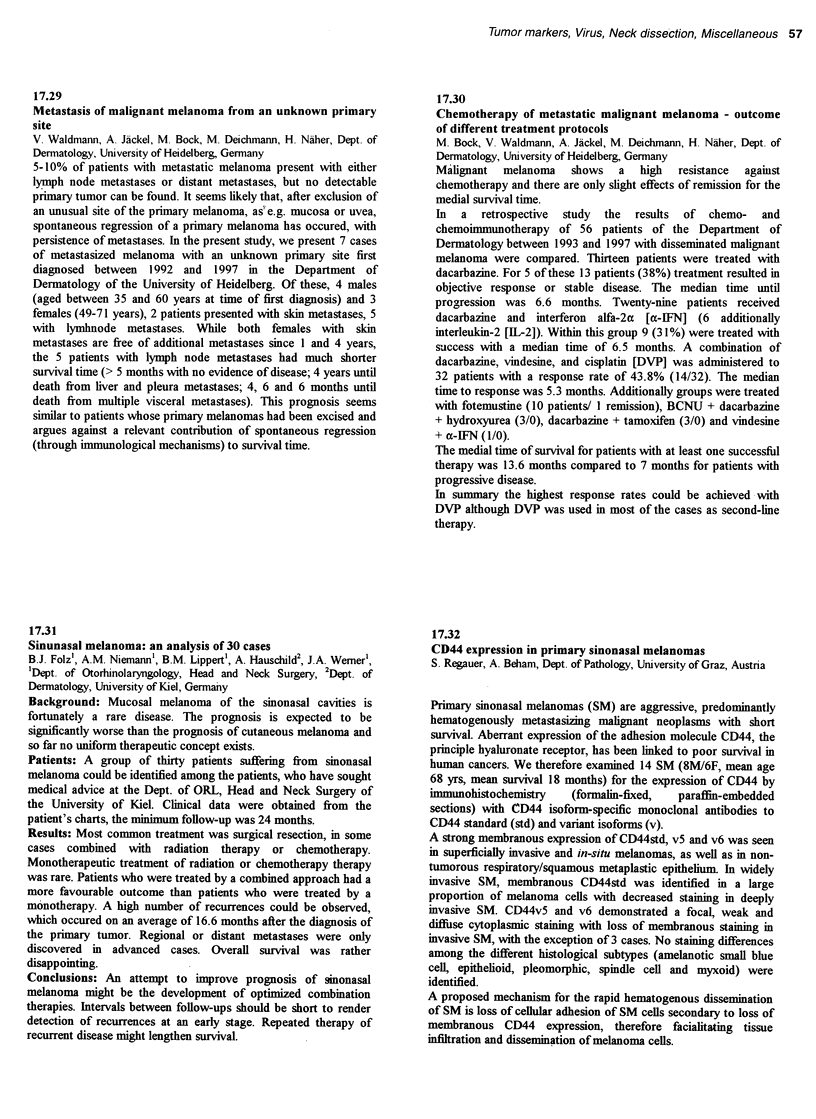# Tumour markers, Virus, Neck dissection, Miscellaneous

**Published:** 1998

**Authors:** 


**Human papillomaviruses in neck metastases of squamous cell carcinoma of the head and neck**

M. Hoffmann[1], T. Kahn[2], C.G. Mahnke[1], B.M. Lippert[1], T. Görögh[1], J.A. Werner[1], [1]Dept. of ORL, University of Kiel, [2]German Cancer Center, Heidelberg, Germany

Applying the most accurate HPV detection methods presently available a HPV prevalence of about 26% can be expected in squamous cell carcinomas of the head and neck, as we have shown earlier. In this study, we investigated the presence of detectable HPV DNA in neck metastases (NM) of this tumor entity by the same methods. Furthermore, in eight cases the correlation of primary tumor (PT) and NM of the same patients could be analyzed.

„Hot start" polymerase chain reaction employing type specific primers as well as consensus primer pairs followed by Southern blot hybridization of the PCR products was performed on DNA extracted from fresh frozen material of 27 NM and 9 PT samples.

HPV DNA was detectable in 13/27 (48%) NM specimens and in 4/9 (44.3%) PT specimens. HPV 16 could be identified in 5/13 (38.8%) HPV positive NM specimens and in 3/4 (75%) positive PT specimens; in nine cases the infecting HPV type could not yet be identified. In 3/4 (75%) HPV positive PT cases the investigated NM samples showed detectable HPV DNA as well, and in 4/5 (80%) cases both NM and PT samples of the same patients were HPV negative.

The prevalence of HPV DNA in PT of patients that develop resectable NM seems to be higher than in those that do not, though the high prevalence rate might also be due to the small number of cases investigated. NM show detectable HPV DNA in 48% of the cases reflecting an association of HPV infection and progression to metastases. The presence of HPV 16 DNA in 38.8% of the positive NM cases points to a special role of this „high risk" HPV type also in developing NM. The high rate of HPV positives that could not be identified might be due to integration of the viral DNA into the genome thought to be a further sign of tumor progression. The strong correlation of the HPV status in PT and NM of the same patients (77.8%) supports the hypothesis that HPV specifically infects the tumor cell.

### 17.2

**p53 immunoreactivity independent of TNM status and HLA-class I in larynx cancer patients?**

S. Gottschlich, E. Westphal[1], I. Rathcke, B.M. Lippert, T. Görögh, T. Wilms, J.A. Werner, Dept. of ORL, [1]Dept. of Immunology, University of Kiel, Germany p53 antibodies are a fairly new serological parameter in cancer patients to determine the p53 status of these patients. The actual mechanism and cause why p53 reaches immunogenicity is unknown, but several studies suggest a prognostic value for p53 antibodies in cancer patients. Ninety-five larynx cancer patients were investigated for their p53 antibody status with ELISA and their HLA class I was determined. The cancer patients were staged according to the TNM-system (N0 n=47, N1 n=19, N2 n=29; M1 n=2) of the UICC. Eighteen of the patients (18.9%) were seropositive for p53 antibodies. No correlation between the NM status and HLA class I of the larynx cancer patients and their p53 antibody status was observed. These preliminary results seem to indicate that the p53 immunoreactivity is independent of the HLA class I in larynx cancer patients and that p53 antibodies do not indicate a higher probability of a primary larynx carcinoma to metastasize.

### 17.3

**The degree of cell surface sialylation of metastases of head and neck cancer**

F. Riedel, W. Bergler, A. Baker-Schreyer, K. Götte, G. Petroianu, K. Hörmann, Dept. of ORL, Mannheim, University of Heidelberg, Germany

**Purpose:** The sialoglycans and the degree of sialylation on the cell surface are of increasing interest because of the possible contribution to metastasis and invasion. Primary tumors and metastasis may differ in the degree of sialylation.

**Material and methods:** Cell surface sialylation of 30 lymph node metastases and 30 squamous cell carcinomas of the head and neck was determined by a new histobiochemical assay on cryostat sections which is based on the enzymatic introduction of a fluorescence-labelled sialic acid (CMP-9-fluoresceinyl-NeuAc) into lactosaminyl type (Gal β 1-4 GlcNAc) oligosaccharide chains of cell surface glycoproteins by α-2,6-sialyltransferase. To compare degree of sialylation with the total amount of sialylation sites a pretreatment with sialidase for desialylation was required.

**Results:** We observed a significant (p=0.001) higher amount of lactosaminyl type binding sites for sialic acid on metastases compared to the primary tumors. Pretreatment with sialidase could detect a significant amount of sialylation on primaries and metastases, showing a lower degree of sialylation in metastases. In primary tumors no correlation was seen between the amount of binding sites and tumor localization, TNM-stage or histological grading of the tumors.

**Conclusions:** A higher degree of sialic acid free lactosaminyl glycans on the cell surface of head and neck tumors seems to be correlated with the occurence of metastasis. Our new histobiochemical method turned out to be effective, reliable and little laborious.

### 17.4

**Expression of metalloproteinases and their inhibitors in squamous cell carcinoma cell lines of the head and neck**

H.G. Schulz, T. Görögh, J. Bäumken, S. Höft, J. Stöhlmacher, B.M. Order, J.A. Werner, Dept. of ORL, University of Kiel, Germany

**Background:** Matrix metalloproteinases (MMP) are a family of proteolytic zinc metalloenzymes implicated in the invasion and metastasis of many cancers. The ability of carcinoma cells to produce extracellular matrix degrading enzymes plays an important role in the malignant behaviour of the neoplasms. **Methods:** Total RNA from 10 laryngeal squamous cell carcinoma (SCC) cell lines with different metastatic potential and 5 keratinocyte cell lines was reverse transcribed and then amplified by polymerase chain reaction (RT-PCR). Specific oligonucleotid primers for MMP-1, MMP-2 and TIMP-1, TIMP-2 were used for amplification. **Results:** The results showed the presence of MMP-1 positive in 100% of the SCC's and 80% of the keratinocytes. MMP-2 was positive in 100% of the tumor cell lines and 80% of the keratinocytes. The inhibitor TIMP-1 was detected in 40% of the SCC's and 60% of the keratinocytes whereas inhibitor TIMP-2 was found positive in 50% of the tumors and 80% of the keratinocytes. The results showed gene expression of MMP's more often in tumor cell lines whereas gene expression of their inhibitors TIMP's was detected more frequently in benign keratinoyte cell lines. **Conclusions:** Our findings suggest that the MMP-TIMP profile may be a tool for prognostic evaluation of the malignant potential of a tumor. Although there are differences in gene expression of MMP's and TIMP's in tumor cell lines and keratinocytes, further investigations with regards to tumor invasiveness and lymph node metastases will be necessary for understanding the role of MMP's and TIMP's in the emergence of cancer.

### 17.5

**MMP and TIMP mRNA expression in head and neck squamous cell carcinomas: a tentative correlation with metastatic potential?**

J. Bäumken, B.M. Lippert, H.G. Schulz, B.J. Folz, M. Seiwerts, E. Weber, J.A. Werner, Dept. of ORL, University of Kiel, Germany

**Background:** Matrix metalloproteinases (MMP) are proteolytic enzymes capable of degrading tissue components. Meanwhile there is some evidence that divergence in the secretion of MMP's or alterations in the differential control by their tissue inhibitors of metalloproteinases (TIMP) play a crucial role in the emergence of metastases. **Methods:** Gene expression of MMP-1, MMP-2, and TIMP-1, TIMP-2 was quantitative analyzed by reverse transcription-PCR in 48 specimen of head and neck squamous cell carcinomas (HNSCC) with different metastatic potential. **Results:** Expression of MMP-1 could be detected in 20,8% of the tumors with and in 45,8% without lymphnode metastases. Within all tumors with positive lymphnodes MMP-2 was expressed in 95,8% whereas MMP-2 was expressed in 45,8% with negative lymphnodes. TIMP-1 was found positive in 45,8% of the tumors with positive and in 83,3% of the tumors with negative lymphnodes. TIMP-2 was positive in 4,2% of the tumors with and in 50% of the tumors without lymphnode metastases. We found a correlation between the expression of MMP-2 and inhibitor TIMP-2 and a weak correlation between the expression of MMP-1 and inhibitor TIMP-1 and the occurrence of lymphnode metastases. **Conclusions:** To our knowledge this is the first study, that shows a correlation between MMP- and TIMP-expression and the metastatic potential of HNSCC using this method. Final statements will only be possible with the investigation of a greater number of patients and consideration of further clinical parameters, e.g. grading and tumor location.

### 17.6

**New definition of the tumor margin by the expression of EP-CAM**

M. Andratschke, S. Chaubal, S. Lang, R. Zeidler, B. Wollenberg, Dept. of Otorhinolaryngology, LM University of Munich, Germany

The epithelial glycoprotein 40 (EGP40), also known as Ep-CAM, is a transmembrane glycoprotein that is expressed on the baso-lateral surface of many human epithelia. The exact function of the molecule is still unknown, but the molecule is involved in Ca++-independent intercellular adhesion. The protein is expressed by 98% of the squamous head and neck carcinomas and is absent in normal squamous epithelia. The aim of our study was the expression of Ep-CAM in the marginal zone of the tumor. Probes of primary HNSCC, epithelium 5mm close to the tumormargin and healthy epithelium were taken intraoperatively and snapfrozen in liquid nitrogen. An RT-PCR was performed with primers for Ep-CAM. The main tumor showed high expression of Ep-CAM. This expression gradually diminishes by enlarging the distance to the tumor margin and becomes negative in healthy mucosa. Future immunhistochemical staining results will correlate this finding with the degree of dysplasia along the tumor margin and its surrounding tissue. Equivalent to the findings of p53-mutations along the tumor border in HNSCC, Ep-CAM expression could be a new marker for redefining the real tumor margin, urging the need to also resect dysplastic tissue.

### 17.7

**Is telomerase activity a possible marker for the metastasis of head and neck cancer?**

S. Koscielny, W. Fiedler*, R. Dahse*, J. Sonntag*, S.M.E. Hofmann*, U. Claussen*, E. Beleites, G. Ernst*, Dept. of ORL, *Inst. of Human Genetic and Anthropology, University of Jena, Germany

Telomerase, the enzyme which is able to stabilize the telomeres of the chromosomes, is closely associated with the immortalization of cells.

In order to evaluate whether the determination of telomerase activity is a possible prognostic marker for the metastatic potential of head and neck cancers we investigated 37 squamous cell carcinomas (SCC) of the oropharynx, 12 SSC of the hypopharynx, 25 SSC of the larynx and 4 SCC of the oral cavity. In 29 cases with oropharyngeal/ 15 with laryngeal/ 5 with hypopharyngeal/ 2 with oral cavity SCC the telomerase status in the corresponding lymph nodes were tested, too.

Approximately 75% of the primary tumors were found to be telomerase positive. In all histological positive nodes we detected telomerase activity. Interestingly, telomerase activity was also present in 90% of the histological negative lymph nodes. The latter may be due to activated lymphocytes which are known to be telomerase active.

By correlating clinical and molecularbiological datas we will discuss our conclusion that the detection of telomerase activity in primary head and neck tumors is not necessarily a reliable predictor for the metastastic potential of these tumors.

### 17.8

**Proliferative activity, determined by the monoclonal antibody KiS11, in correlation to survival in patients with lymph node metastasis of hypopharyngeal squamous cell carcinoma**

C. Kuropkat, R.W. Huang, M. Hoffmann, R. Parwaresch, B.M. Lippert, J.A.Werner, Rush Univ. Chicago, USA, Univ. of Kiel, Germany

**Introduction:** Recently, we found a significant correlation between the immunohistochemically determined proliferation status and the survival of patients with squamous cell carcinoma of the hypopharynx (SCCH). The aim of this study was to verify the same relationship for the lymph node metastasis (LNM) of these tumors. Additionally we compared the proliferation indices of primary tumor and LNM. **Material and methods:** In a retrospective study we immunohistochemically investigated the proliferative activity of paraffin-embedded LNM of 49 patients with primary SCCH. We used the monoclonal antibody Ki-S11, which recognizes the Ki-67-antigen. The 5-year-survival was calculated according to the Kaplan-Meier-technique. **Results:** Patients with low ($\leq$45%) proliferating LNM had a significantly higher 5-year-survival-rate (44%) than those with high (>45%) proliferation LNM, who had a 5-year-survival-rate of 29% ($p<0.05$). The LNM had a median proliferation rate of 52% in correlation to 49% for the primary SCCH ($p<0.223$). **Conclusion:** There is no significant difference in the proliferation rate of primary SCCH and their LNM. Patients with high proliferating LNM have a less favorable clinical outcome compared to those with low proliferating LNM. The antibody Ki-S11 seems to be a potential tool to determine the proliferative activity and to predict patient survival in primary SCCH as well as in their LNM.

### 17.9

### Proliferative activity in early stages of metastases

M. Lörz, Bremervörde, Germany

A possible technique to study metastasis is to inject tumor cells intravenously into healthy animals and to follow them up until metastases arises. Proliferation in metastases can be determined by combining the methods of autoradiography (Lörz, M. et al. 13th meeting ESGCP, October 1984) and immunocytochemistry (monoclonal antibodies).

Tritiated-thymidine (3H-TdR) tumor cells were injected into the tail vein of adult, syngenic Sprague-Dawley cure rats and resulted in the development of lung metastases. Arrest of disseminated tumor cells in a target organ does not necessarily result in formation of metastases. Therefore a second label with 14C-TdR was administered at different intervals after implantation of pre-labelled tumor cells. The combination with antiproliferative monoclonal antibodies reveals the possibility for further investigations.

Despite the high rate of proliferation of most disseminated cells shortly after arrest in the target organ, only few clones developed into progressive metastastic foci indicating an inherent proliferative and metastatic heterogenity.

### 17.10

### Follow-up of patients with carcinoma of head and neck using the tumor marker Cyfra 21-1 for early detection of recurrent disease

I. Doweck, M. Barak, N. Uri, E. Greenberg, Dept. of ORL, Biochemistry Research Unit, Carmel Medical Center, Haifa, Israel

Cyfra 21-1 was reported as a new potential tumor marker for squamous cell carcinoma of head and neck. In the present study we evaluated the additional value of Cyfra 21-1 in the follow up of patients with head and neck cancer. The study included 38 patients with primary detection of head and neck carcinoma, who were followed up for at least one year during the different treatment regimens. Patients were followed every month for the first year after diagnosis and every three months for the following years. Cyfra 21-1 was determined periodically using an Elisa kit. Clinical suspicion of recurrence or a significant increase in the Cyfra 21-1 level (>25%) was further investigated by histological biopsy.

Thirty eight patients were followed using Cyfra 21-1. Out of 38 patients, 12 had recurrent disease. In 10 of these patients (83%), Cyfra 21-1 was elevated before or concomitantly with the clinical detection of the recurrence. 26 patients had no clinical evidence of disease during the evaluation. In 20 of them (77%), there was no significant elevation in the marker levels during follow up. Elevated levels of the marker without clinical evidence of disease can be attributed to the mean lead time of the marker as compared to clinical appearance of the disease.

**Conclusion:** Cyfra 21-1 was found as an additional useful tool for the follow up of patients with carcinoma of head and neck.

### 17.11

### Cyfra 21-1: an indicator for distant metastases in head and neck cancer?

J.D. Maass, A.M. Niemann, S. Gottschlich, B.M. Lippert, A. Oelerking, J.A. Werner, Dept. of Otorhinolaryngology, Head and Neck Surgery, University of Kiel, Germany

**Introduction:** Patients with head and neck squamous cell carcinoma (HNSCC) are usually followed-up clinically. During the follow-up it is difficult to detect distant metastases at early stages, while a locoregional tumor progression sometimes can be seen quite easily. Cyfra 21-1, a novel tumor marker, was shown to correlate with tumor size in HNSCC. Therefore, it seemed worthwhile elucidating a possible role of cyfra 21-1 in the metastastic progression of HNSCC. **Methods:** During the follow-up, we compared the course of cyfra 21-1 values in sera of HNSCC patients with distant metastases (n=18) and without distant metastases (n=310). **Results:** 17 of 18 patients with distant metastases had cyfra 21-1 values above the threshold of 2.2 ng/ml. Initially high or suddenly rising cyfra 21-1 values during the follow-up mostly preceded or coincided with the detection of distant metastases. **Conclusions:** The presented results indicate that cyfra 21-1 may be useful in the early diagnosis of distant metastases in HNSCC. This may influence therapeutical decisions that sometimes go along with disfiguring surgical procedures, e.g. laryngectomy. The early detection of distant metastases may avoid unnecessary suffering for these patients, who may then profit from an early cooperation between different clinical departments and alternative therapy plans.

### 17.12

### Diagnosis and management of distant metastasis of the carcinoma of the oropharynx and larynx - experience from 1980 - 1990

S. Koscielny, J. Stein, B. Kratsch, Dept. of Otorhinolaryngology, University of Jena, Germany

Among other things the long time survival of patients with a head and neck cancer is determined by the occurrence of distant metastasis. Because of this fact we were interested in the rate of distant metastasis by patients with a squamous cell carcinoma of the oropharynx and larynx over 15 years from 1975 to 1990. All patients were observed in our outpatient department at least 5 years after diagnosis of their tumor disease.

**Patients:** 163 patients with a carcinoma of the oropharynx and 170 patients with a carcinoma of the larynx were examined. The number of distant metastasis and their management were analysed.

**Results:** In 11 (6,7%) of the patients with a laryngeal cancer (7 supraglottic and 4 glottic) we observed distant metastasis. It's interesting that 8 of these patients had an N0-neck during the time of tumor therapy. Among the patients with oropharyngeal cancer 18 (10,6%) developed distant metastases in course.

Most of all distant metastases were located in the lungs. Over all we observed three abdominal metastases and one metastasis of the spine.

### 17.13

### Cystically degenerated neck node metastases mimicking malignoma in lateral cervical cysts

B.J. Folz, A.M. Niemann, B.M. Lippert, ddJ.A. Werner, Dept. of Otorhinolaryngology, Head and Neck Surgery, University of Kiel, Germany

There is a lot of controversy about the existence of malignant dedifferentiation of lateral cervical cysts. More likely than a primary malignoma in a branchial cyst is the occurrence of a cystically degenerated metastasis to the neck. The differentiation between these two entities is of clinical importance for the patient in terms of diagnostic measures and therapy. Between 1990 and 1996 pathology reports revealed a squamous cell carcinoma in 5 patients who were clinically primarily diagnosed lateral cervical cyst. One female patient was diagnosed malignant melanoma in a branchiogenic cyst. On ultrasound examination the preoperative findings had not been different from "normal" cervical cysts. To rule out metastatic disease all 6 patients underwent a staging consisting of panendoscopy with tonsillectomy and lasersurgical resection of the lingual tonsil. Histologic examination of the specimen revealed a primary cancer in 3 patients (lingual tonsil: 2 cases, palatine tonsils: 1 case). Clinically the areas had not been suspicious for malignancy. One further patient developed a carcinoma in his base of tongue within a year after initial diagnosis. These findings indicate that metastatic disease should rather be considered than a primary malignancy of a lateral cervical cyst. Still malignant dedifferentiation in lateral cervical cysts does occur and histologic diagnosis of carcinoma in situ or severe dysplasias confirm the lateral cervical cyst as the primary lesion. However it should be recommended that these patients are referred to a complete staging including multiple biopsies to exclude a metastasing primary malignancy elsewhere in the upper aerodigestive passage, especially as there appears to be a predisposition for carcinomas originating from Waldeyers Ring to form cystic metastases to the neck.

### 17.14

### Cystic carcinoma in cervical lymph nodes express a different cytokeratin profile than benign lateral neck cysts

S. Regauer, A. Beham, Dept. of Pathology, University of Graz, Austria

"Branchiogenic carcinomas" (=cystic carcinomas located within cervical lymph nodes) are metastases from squamous cell carcinomas (SCC) of head and neck, especially of Waldeyer-ring origin. We compared the cytokeratin (CK) profile of 15 cystic cervical lymph node metastases and their primary carcinomas (10 tonsils, 5 base of tongue) with that of benign lateral neck cysts (LNC).

All lymph nodes contained poorly differentiated cystic malignant sqamous epithelium (0.5 - 5m diameter) without transitions from non-tumorous to dysplastic or frankly malignant epithelium with uniform expression of "simple" CK 8 and 19 throughout the cystic carcinoma but only very focal "simple" CK7 and "stratified-epithelial-type" CK13 expression. The CK-profile of the primary SCC was identical to that of the cystic carcinoma in lymph nodes. Expression of CK 4, 5, 10, 14, 17 and 18, typically present in LNC, was not observed in either primary or metastatic carcinoma.

The CK-patterns of metastatic cystic SCC and primary SCC of Waldeyer's ring origin are identical but distinctly different from the CK-profile of benign LNC, which is characterized by an orderly and differential expression of "simple" CK in basal cells and "stratified-epithelial-type" CK in suprabasal cells (APMIS, 105, 623-630, 1997).

### 17.15

### Value of elective neck dissection as a staging procedure in patients with head and neck cancer

S. Höft, S. Eggers, B.M. Lippert, M. Weller, J.A. Werner, Dept. of Otorhinolaryngology, Head and Neck Surgery, University of Kiel, Germany

**Introduction:** Lymph node metastases are often difficult to detect. However, if present - detected or undetected - they severely reduce the patient´s chances of healing. A controversy evolves around the usefulness of elective neck dissection. The aim of the present study was to determine how often the posttherapeutic N-status had to be changed due to a histopathological diagnosis differing from $N_0$. In this respect the validity of the imaging procedures in staging was investigated.

**Patients and methods:** From April 1996 until May 1997 12 patients with squamous cell carcinomas of the head and neck underwent an elective neck dissection. The tumors were located in the oral cavity (3), oropharynx (2), hypopharynx (1), and larynx (6). All patients were staged with ultra sound. Additionally 7 patients had a CT, 4 an MRI, and 1 patient had a CT and an MRI of the neck.

**Results:** The histopathological diagnosis was N0 in 9 cases, N1 in 2, and N2b in 1 patient.

**Conclusions:** In one quarter of the patients lymph node metastases were present that had not been detected in preoperative staging. The study supports the point of view of different centers that an elective neck dissection is either a staging method or in a considerable number of patients a therapy.

### 17.16

### Neck lymph node biopsy did not increase the distant metastases in AJCC $T_{1-3}N_{1-3}$ nasopharyngeal carcinoma patients

P.C. Pai[1], N.M. Tsang[1], C.F. Lin[2], C.K. Tseng[1], S. Tang[1], [1]Dept. of Rad. Oncology, [2]Dept. of Public Health, Chang Gung College of Medicine and Technology, Linkou, Taiwan

**Purpose:** To investigate whether the neck lymph node biopsy procedure increases the distant metastases rate in AJCC $T_{1-3}N_{1-3}$ nasopharyngeal carcinoma (NPC) patients. **Material and Methods:** From February 1979 to December 1991, 1181 NPC patients were treated in Chang Gung Memorial Hospital radiation oncology department. These patients completed the proposed course of nasopharyngeal treatment equal or greater than 64 Gy and were followed up for more than 5 years. Five hundred and forty-three patients belonged to AJCC $T_{1-3}N_{1-3}$ stage and in which 88 cases had neck lymph node operation procedure (aspiration 7, incision 39, excision 40, and dissection 2). **Results:** No statistical difference is recognized in age, operation method, or the time interval between operation and start of local treatment but sex, N stage, and neck area electron boost. **Discussion:** In this study, though it is suggested that neck lymph node operation before local-regional irradiation do not increases the distant metastatic rate in AJCC $T_{1-3}N_{1-3}$ NPC patients.

### 17.17

**Neck lymph node metastases affecting the distant metastases in AJCC $T_4$ nasopharyngeal carcinoma patients**

C.-K.Tseng[1], N.-M. Tsang[1], S.-K. Lo[2], [1]Dept. of Radiation Oncology, [2]Dept. of Public Health, Chang Gung College of Medicine and Technology, Linkou, Taiwan

**Purpose:** To study the risk factors affecting the distant metastases in AJCC $T_4$ nasopharyngeal carcinoma (NPC) patients. **Material and Methods:** From February 1979 to December 1991, 1181 NPC patients were treated in Chang Gung Memorial Hospital radiation oncology department. These patients completed the proposed course of nasopharyngeal treatment equal or greater than 64 Gy and were followed up for more than 5 years. Four hundred patients were $T_4$ cases (skull base destruction, brain invasion, retrobulbar invasion, cranial nerve palsies or sphenoid sinus invasion). **Results:** N stage is the only factor affecting the distant metastases in AJCC $T_4$ NPC patients (p=0.0001) after local complete irradiation. Age, sex, pathology, sites of tumor involvement, cranial nerve palsies, treatment interval, electron neck boost dose or nasopharynx brachytherapy did not affect the outcome of AJCC $T_4$ NPC patients. **Conclusion:** In this study, the higher the N staging, the higher the probability of distant metastases are recognized in AJCC $T_4$ NPC patients. It suggests that the NPC treatment outcome of $T_4N_0$ NPC patients is different from $T_4N_3$ though all belong to stage 4 in AJCC staging.

### 17.18

**Metastatic spread of adenoid cystic carcinoma of the salivary glands - A retrospective analysis of 50 cases**

C.G. Mahnke, C. Külkens, J. Preugschat, C. Wenzel, S. Saffran, J.A. Werner, Dept. of Otorhinolaryngology, Head and Neck Surgery, University of Kiel, Germany

Among salivary gland malignancies adenoid cystic carcinoma (ACC) differs in that it often leads to distant metastasis. The aim of this study was to analyse the clinical course of patients diagnosed with ACC with special consideration to metastasis.

The charts of all patients (n=50, m:26, f: 24) treated at the Dept. of Otorhinolaryngology, Head and Neck Surgery, University of Kiel, Germany, between 1954 and 1993 for ACC of the salivary glands were analysed retrospectively. TNM-classification was defined following the guidelines of the UICC (1987).

T and N classification was as follows: T1: 8 (N0: 7, N1: 1); T2: 11 (N0: 10, N1: 1); T3: 15 (N0: 12, N1: 2, N2: 1); T4: 6 (N0: 4, N2: 2). Distant metastasis occured in 13 patients [26% (lung: 9, liver: 1, distant lymphatics: 1, skeleton: 2)]. Two of these patients suffered from metastasis to multiple sites. Diagnosis of distant metastasis was established between 0 and 172 months after diagnosis of the primary tumor (average 63 ± 61 months). Following diagnosis of distant metastasis the average survival was 21 (± 24) months and five patients survived 2 or more years and in some cases significantly longer.

Even though the rate of distant metastasis in ACC of the salivary glands is high patients often survive for many years due to a slow tumor progression at the site of metastasis.

### 17.19

**Lymphoscintigraphy of the neck showing aberrant lymphatic drainage**

S. Höft[1], K.H. Bohuslavizki[2], S. Klutmann[2], E. Henze[2], J.A. Werner[1], [1]Dept. of Otorhinolaryngology, Head and Neck Surgery, [2]Dept. of Nuclear Medicine, University of Kiel, Germany

**Introduction:** Lymphoscintigraphy is a standard procedure for detecting sentinel lymph nodes in patients with malignant melanoma. We evaluated patients with squamous cell carcinomas of the head and neck.

**Patients and Methods:** From August 1996 until June 1997 46 patients with 47 squamous cell carcinomas of the head and neck were injected 50-100 MBq Tc-99-m-colloid peritumorally. 20 minutes later 50 MBq Tc-99-m-pertechnetate were injected intravenously to picture the body contours. The nodal status of the tumors was N0=20, N1=7, N2a=2, N2b=5, N2c=12, N3=1. 42 patients were scanned at 30 min and 4-6 hours after injection. 4 patients were scanned 24 hours after they had been injected within the staging panendoscopy due to the localisation of the tumor.

**Results:** In 11 cases no drainage was observed. 23 patients had an ipsilateral and 12 patients with an unilateral tumor had an bilateral (n=9), contralateral (n=1) or retropharyngeal (n=2) drainage. Of these patients 2 had lymph node metastases bilaterally. Thus, in 10 tumors (21%) with ipsilateral metastases an unexpected lymphatic drainage concerning the retropharyngeal and contralateral lymph nodes was observed. One patient with a midline crossing tumor had a drainage on the less affected side.

**Conclusion:** Cervical lymphatic drainage could not be predicted. Further investigation has to be directed into evaluating sentinel lymph nodes and their value of predicting metastatic disease in the neck.

### 17.20

**Selective neck dissection versus observation in the case of T1 and T2 oral carcinoma**

R. Schimming, U. Eckelt, Dept. of Oral and Maxillofacial Surgery, University of Dresden, Germany

The purpose of this investigation was to compare selective neck dissection (SND) versus observation for the surgical treatment of T1 and T2 squamous cell carcinoma of the oral cavity (SCCOC) with clinically negative (N0) neck.

162 patients (70-T1/92-T2), treated surgically for SCCOC, entered this retrospective study between 1974 and 1994. 57 patients (12/45) underwent SND simultaneously with their surgery for the primary tumor. 105 patients (58/47) did not receive neck surgery. The outcomes measured include local recurrence, regional metastasis, distant metastasis, failure free survival (FFS) and overall survival (OS).

SND did not reduce the local recurrence rate (chi-squared test, p = 1.0) but did significantly diminish the regional metastasis rate (chi-squared test, p = 0.04). The regional metastasis related mortality was significantly decreased (chi-squared test, p = 0.03) for patients treated with SND. No significant correlation was observed in the case of FFS (Log-rank test, p = 0.7) as well as OS (Log-rank test, p = 0.7).

This study establishes that SND has a greater benefit in the treatment of T1 and T2 SCCOC with clinically negative (N0) neck than observation.

### 17.21
### Different incisions to approach neck dissections

[1]M.C. Heissenberg, [1]S. Höft, [1]B.M. Lippert, [2]G.M. Zotter, [1]J.A. Werner, [1]Dept. of Otorhinolaryngology, Head and Neck Surgery, University of Kiel; [2]Berlin, Germany

The idiom neck dissection (ND) for surgical approach to cancer disease in the head and neck was first used by G.W. Crile in 1906. Beside the development and evolution of surgical techniques like i.e. modified, radical, supraomohyoidal ND several kinds of incisions to different and, in oncological respects, identical NDs are established and discussed.

In our department we use a small variety of incision techniques depending on tumor location, preoperative TNM-status, preoperative treatment like radiation, and possible reconstructive surgery.

In the majority of cases the "hockey-stick-incision" is used. It offers a wide exposure of the neck giving safe access to the compartment II-VI, the occipital skull base and even most parts of the posterior neck. In cases requiring extended posterior ND, the incision can be extended into a "book incision" as described by E.D. Diaz et al. giving excellent access to all areas of risk. Generally, compartment I is safely approachable by consequent skin retraction. In rare, individual cases the head and neck surgeon could still extend the access into a modified de-Quervain-incision. There is no common risk like tip necrosis as for instance using Y-incisions. U-incisions are best used in patients who will receive a total or partial laryngectomy combined with ND. A MacFee-incision with its broad skin flap probably offers the best skin healing and is discussed for patients with peripheral vascular disease or after radiation therapy. We prefer MacFee-incisions in patients who, after tumor removal, will need reconstructive surgery like free myocutaneous flaps.

In conclusion we think that for most NDs the "hockey-stick-incision" offers safe access to all areas of risk and gives opportunity to extend the incision if needed.

### 17.22
### The preservation of the spinal accessory nerve in case of cervical metastases

J. Mrowetz, I. Karapantzos, R. Kehl, G. Kölmel, Dept. of ORL, Marienhospital Stuttgart, Germany

The only indication to sacrifice the spinal accessory nerve during neck dissection, similary to the hypoglossal nerve, is the certain infiltration by metastases. To ascertain whether there is infiltration of the nerve, it must not be sacrified „on principle“, but it must be explorativly dissected - and only be resected in case of metastatic infiltration - from the oncologically inconspicuous area to the metastatic area. Most frequently the dissection is carried out in caudocephalad direction, for most cervical metastases occur in the upper third of the neck. The method of uncovering the accessory nerve in the supraclavicular area will be described by the authors.

Our observations for more than 10 years have not shown any difference concerning the frequency of metastatic recurrence in the area of the spinal accessory nerve between preserving and sacrifycing the nerve.

A permanent failure is not to be expected when the nerve is prepared carefully, as done in the surgery of the parotid gland.

### 17.23
### Clinical experience with intratumoral injection of cisplatin/epinephrine gel in patients with tumor recurrences or regional metastases in head and neck cancer

C. Külkens, S. Höft, B.M. Lippert, J.A. Werner, Dept. of ORL, University of Kiel, Germany

**Background:** A new approach to treat patients with lymph node metastases in the neck due to recurrent squamous cell carcinoma of the head and neck (SCCHN) is using cisplatin/epinephrine gel (MPI 5010) for intratumoral injection.

**Patients and Methods:** Fifteen patients with recurrent SSCHN have been included into a currently ongoing multicentre, randomised, double-blind and placebo-controlled study at the Department of Otorhinolaryngology, Head and Neck Surgery, University of Kiel since June 1996. Twelve patients were treated for local recurrences, three patients had regional lymph node metastases in the neck which were treated by intralesional injections using cisplatin/epinephrine gel.

**Results:** These three patients did not receive 6 injections according to protocol because of disease progression at the target tumor or systemic disease progression (i. e. development of distant metastases). Two patients developed lung metastases and were switched to systemic chemotherapy after the third injection. As a result one patient had a complete remission. The third patient's general state of health deteriorated rapidly due to tumor progression. Therefore, treatment was abandoned after the first injection. In 3 of 15 patients treated for local reurrences no tumor could macro-/microscopically be detected.

**Conclusion:** Intralesional chemotherapy is a new well tolerated and feasible palliative approach to treat selected patients with recurrent SSCHN. Intratumoral administration of cisplatin/epinephrine gel in patients with large metastases however may cause complications. Treatment responses which were observed in local recurrences could not be observed in lymph node metastases. Further clinical studies will demonstrate the importance of this new therapeutic approach.

### 17.24
### Prognostic evaluation of squamous cell carcinoma of the oral cavity

T. Bundgaard, Dept. of Otolaryngology, University of Aarhus, Denmark

**Introduction:** Prognostic indicators that could assist in a more precise selection of patients with oral cancer for diffentiated therapy would be clinically valuable.

**Methods:** A consecutive series of 161 cases of intraoral squamous cell carcinoma (SCC) occurring during a 5-year period in a population of 1.4 million inhabitants, was evaluated by histopathologic, stereologic, clinical, and epidemiologic parameters.

**Results:** Univariate analysis established a significant prognostic value in terms of cause-specific survival for T stage, maximum tumor diameter, N stage (N+/N0), alcohol consumption, sterologic estimates of nuclear volume, and the histomorphologic parameters mode of invasion, pattern, vascular invasion, depth, and mean histologic score. Tobacco consumption was borderline significant. A multivariate analysis using the Cox proportional hazard analysis showed that both clinical (stage, $P<0.0001$; size, $P=0.0027$), epidemiologic (tobacco consumption, $P=0.0054$), morphohistopathologic (mode of invasion $P<0.0001$), and stereologic (nuclear volume, $P=0.0010$) parameters had an independent significant effect on survival. Twelve patients (25%) with stage I disease had unfavorable histologic and stereologic parameters. The observed survival for these patients was 33% ± 18%. The observed survival for stage I patients with more favorable histologic characteristics (n=36) was 76% ± 8%.

**Conclusion:** The use of a combination of clinical, histological, epidemiologic, and stereologic parameters will assist the design of treatment strategies for intraoral SCC including treatment of the neck.

### 17.25

**Importance of the N-status for prognosis and treatment in glottic cancer patients**

J. Büntzel[1], M. Glatzel[2], D. Fröhlich[2], K. Küttner[1], [1]Dept. of ORL, Plastic Surgery, [2]Dept. of Radiotherapy, Clinic of Suhl, Germany

**Objective:** Besides the well known influence of lymphatic metastasis on the follow-up and the prognosis of glottic cancer, there are different opinions about the importance of this fact on treatment decisions.

**Material and methods:** We analyzed the data of 178 patients with glottic cancer who were treated at our departments between 1990 and 1995.

**Results:** The incidence of N-positive disease (clinical picture, MRI or CT or sonography) was 20,7%. It was dependent from the size of primary tumor: T2 - 11,8% N+disease, T3 - 37,0% N+disease, T4 - 52,0% N+disease. The 5-years-survival-rates differed significantly between N0- and N+disease: 86% versus 59%. In the case of lymphatic metastasis of glottic cancer our standard treatment (64,9%) was a neck dissection of the lymph-node positive side, followed by an adjunctive radiotherapy (60 Gy total dose, 2 Gy single dose, daily fraction). 13,5% of all patients were treated by primary radiochemotherapy (simultaneous in combination with carboplatin). 21,5% of N+patients wished to be treated only by surgery. If the N+patients got the standard treatment the 5-years-survival-rate was 62% compared to 45% following surgical treatment alone.

**Conclusion:** The treatment of advanced glottic cancer with lymphatic metastasis should include the microscopic resection of the primary cancer, followed by the neck dissection and the largefield radiation therapy of the cervical lymph nodes.

### 17.26

**A clinico-pathological examination of parastomal neoplasm etiology of laryngeal carcinoma**

F.U. Metternich, T. Brusis, Dept. of Otorhinolaryngology, Head and Neck Surgery, Clinic Holweide, Cologne, Germany

**Background/Patients:** Parastomal neoplasm after total laryngectomy for laryngeal carcinoma represents an extremely serious complication. To clarify the controversial aspects of parastomal neoplasm etiology, a systematic analysis was performed using data from 2680 patients with total laryngectomy. **Results:** Parastomal neoplasm occured in 6.4%. The tumour site of the primary laryngeal carcinoma was found in 76.1% in the sublottic or supraglottic area. If the laryngeal carcinoma was resected with margins of healthy tissue, lymphatic metastasis to the pretracheal and paratracheal cervical nodes is the probable cause of parastomal neoplasm. The cervical metastasis formation cannot be detected due to the limitations in the assessment of small lymph nodes in radiologic imaging and due to the limitations in the removal of lymph nodes in the pretracheal and paratracheal area by means of a functional or radical neck dissection. This could be the consequence of the continuous lymphatic drainage between the supraglottic and subglottic area with a midline crossing and a lymphatic outlet to the pretracheal and paratracheal cervical lymph nodes. **Conclusions:** In cases of a patulous subglottic or supraglottic laryngeal carcinoma the method of treatment should be a laryngectomy and a bilateral neck dissection in combination with an ipsilateral and contralateral pretracheal and paratracheal lymph node removal.

### 17.27

**Verification of conformal head and neck irradiation techniques by filmdosimetry**

B. Schicker, U. Götz, I.C. Kiricuta, Inst. of Radiation Oncology, St. Vincenz Hospital, Limburg, Dept. of Biomedical Technique, University of Gießen, Germany

**Purpose:** In case of head and neck cancer we routinely apply conformal 3D - irradiation techniques. Due to the complex beam arrangement we need a tool for quality assurance. In this study we show how calculated dose distributions can be verified by the method of filmdosimetric measurements.

**Material and method:** The measurements are carried out with a humanoid phantom, which is divided up in several transversal slices (Alderson - Phantom). A verification film (Kodak X Omat V) was placed between two phantom slices. The transversal position of the film corresponds to the CT slice in which the dose distribution was calculated. After irradiation the film was developed and scanned with a computer controlled densitometer. The controlling software of the densitometer allows a calibration of the optical densitiy in dose units [cGy].
The dose distribution on the film was compared with the calculated distribution of the planning system.

**Results:** There is a good comprehension between the calculated and the measured dose distributions within a few percent. The determination of the absolute dose in the reference point depends on the exact knowledge of the radiation response curve of the verification film.

**Conclusion:** The filmdosimetry is a simple and quick way to verify the calculated dose distribution. The advantages of the method are:
- Easy to handle
- Quick check routine
- Needs few tools (phantom, filmscanner)
- Usable for all treatment techniques

### 17.28

**Localization of primary tumor correlates significantly with the pattern of initial metastasis in malignant melanoma**

B. Schlagenhauff[1], M.M. Maassen[2], C. Zimmermann[1], U. Ellwanger[1], W. Stroebel[1], G. Rassner[1], C. Garbe[1], [1]Dept. of Dermatology, [2]Dept. of Otolaryngology, University of Tübingen, Germany

At present, only limited information is available of factors influencing the pattern of metastasis in malignant melanoma. Therefore we conducted a study in order to investigate, if the localization of the primary tumor of malignant melanoma had an influence on its pattern of initial metastasis.

Data of 6705 patients with complete follow-up and primary diagnosis from january 1976 until december 1995 were analyzed using the chi-square-test and multiple logistic regression models. Metastasis occured in 960 patients. Distribution of localization of primary tumors (patients with metastases) were: head/neck: 944 (143), trunk: 2521 (400), upper extremity: 1098 (113), lower extremity 2142 (304). Median control period was 40 months.

Results indicated that the incidence of primary satellite/in-transit metastasis was significantly higher in malignant melanoma found in the head and neck region (33,6% of 143 patients) or in the lower extremities (27,6% of 304 patients). Interestingly enough, in malignant melanoma of the head and neck region, incidence of satellite/in-transit metastasis (33,6%), regional lymph node metastasis (37,8%) or distant metastasis (28,7%) was similiar.

As a conclusion, in addition to tumor thickness, localization of the primary tumor of malignant melanoma in the head and neck region, was a factor with impact on the initial pattern of metastasis.

17.29

**Metastasis of malignant melanoma from an unknown primary site**

V. Waldmann, A. Jäckel, M. Bock, M. Deichmann, H. Näher, Dept. of Dermatology, University of Heidelberg, Germany

5-10% of patients with metastatic melanoma present with either lymph node metastases or distant metastases, but no detectable primary tumor can be found. It seems likely that, after exclusion of an unusual site of the primary melanoma, as e.g. mucosa or uvea, spontaneous regression of a primary melanoma has occured, with persistence of metastases. In the present study, we present 7 cases of metastasized melanoma with an unknown primary site first diagnosed between 1992 and 1997 in the Department of Dermatology of the University of Heidelberg. Of these, 4 males (aged between 35 and 60 years at time of first diagnosis) and 3 females (49-71 years), 2 patients presented with skin metastases, 5 with lymhnode metastases. While both females with skin metastases are free of additional metastases since 1 and 4 years, the 5 patients with lymph node metastases had much shorter survival time (> 5 months with no evidence of disease; 4 years until death from liver and pleura metastases; 4, 6 and 6 months until death from multiple visceral metastases). This prognosis seems similar to patients whose primary melanomas had been excised and argues against a relevant contribution of spontaneous regression (through immunological mechanisms) to survival time.

17.30

**Chemotherapy of metastatic malignant melanoma - outcome of different treatment protocols**

M. Bock, V. Waldmann, A. Jäckel, M. Deichmann, H. Näher, Dept. of Dermatology, University of Heidelberg, Germany

Malignant melanoma shows a high resistance against chemotherapy and there are only slight effects of remission for the medial survival time.

In a retrospective study the results of chemo- and chemoimmunotherapy of 56 patients of the Department of Dermatology between 1993 and 1997 with disseminated malignant melanoma were compared. Thirteen patients were treated with dacarbazine. For 5 of these 13 patients (38%) treatment resulted in objective response or stable disease. The median time until progression was 6.6 months. Twenty-nine patients received dacarbazine and interferon alfa-2$\alpha$ [$\alpha$-IFN] (6 additionally interleukin-2 [IL-2]). Within this group 9 (31%) were treated with success with a median time of 6.5 months. A combination of dacarbazine, vindesine, and cisplatin [DVP] was administered to 32 patients with a response rate of 43.8% (14/32). The median time to response was 5.3 months. Additionally groups were treated with fotemustine (10 patients/ 1 remission), BCNU + dacarbazine + hydroxyurea (3/0), dacarbazine + tamoxifen (3/0) and vindesine + $\alpha$-IFN (1/0).

The medial time of survival for patients with at least one successful therapy was 13.6 months compared to 7 months for patients with progressive disease.

In summary the highest response rates could be achieved with DVP although DVP was used in most of the cases as second-line therapy.

17.31

**Sinunasal melanoma: an analysis of 30 cases**

B.J. Folz[1], A.M. Niemann[1], B.M. Lippert[1], A. Hauschild[2], J.A. Werner[1], [1]Dept. of Otorhinolaryngology, Head and Neck Surgery, [2]Dept. of Dermatology, University of Kiel, Germany

**Background:** Mucosal melanoma of the sinonasal cavities is fortunately a rare disease. The prognosis is expected to be significantly worse than the prognosis of cutaneous melanoma and so far no uniform therapeutic concept exists.

**Patients:** A group of thirty patients suffering from sinonasal melanoma could be identified among the patients, who have sought medical advice at the Dept. of ORL, Head and Neck Surgery of the University of Kiel. Clinical data were obtained from the patient's charts, the minimum follow-up was 24 months.

**Results:** Most common treatment was surgical resection, in some cases combined with radiation therapy or chemotherapy. Monotherapeutic treatment of radiation or chemotherapy therapy was rare. Patients who were treated by a combined approach had a more favourable outcome than patients who were treated by a monotherapy. A high number of recurrences could be observed, which occured on an average of 16.6 months after the diagnosis of the primary tumor. Regional or distant metastases were only discovered in advanced cases. Overall survival was rather disappointing.

**Conclusions:** An attempt to improve prognosis of sinonasal melanoma might be the development of optimized combination therapies. Intervals between follow-ups should be short to render detection of recurrences at an early stage. Repeated therapy of recurrent disease might lengthen survival.

17.32

**CD44 expression in primary sinonasal melanomas**

S. Regauer, A. Beham, Dept. of Pathology, University of Graz, Austria

Primary sinonasal melanomas (SM) are aggressive, predominantly hematogenously metastasizing malignant neoplasms with short survival. Aberrant expression of the adhesion molecule CD44, the principle hyaluronate receptor, has been linked to poor survival in human cancers. We therefore examined 14 SM (8M/6F, mean age 68 yrs, mean survival 18 months) for the expression of CD44 by immunohistochemistry (formalin-fixed, paraffin-embedded sections) with CD44 isoform-specific monoclonal antibodies to CD44 standard (std) and variant isoforms (v).

A strong membranous expression of CD44std, v5 and v6 was seen in superficially invasive and *in-situ* melanomas, as well as in non-tumorous respiratory/squamous metaplastic epithelium. In widely invasive SM, membranous CD44std was identified in a large proportion of melanoma cells with decreased staining in deeply invasive SM. CD44v5 and v6 demonstrated a focal, weak and diffuse cytoplasmic staining with loss of membranous staining in invasive SM, with the exception of 3 cases. No staining differences among the different histological subtypes (amelanotic small blue cell, epithelioid, pleomorphic, spindle cell and myxoid) were identified.

A proposed mechanism for the rapid hematogenous dissemination of SM is loss of cellular adhesion of SM cells secondary to loss of membranous CD44 expression, therefore facialitating tissue infiltration and dissemination of melanoma cells.